# Peptide nucleic acid clamp polymerase chain reaction reveals a deletion mutation of the *BRAF* gene in papillary thyroid carcinoma: A case report

**DOI:** 10.3892/etm.2013.1332

**Published:** 2013-10-08

**Authors:** YONG-WHA LEE

**Affiliations:** Department of Laboratory Medicine and Genetics, Soonchunhyang University Bucheon Hospital and Soonchunhyang University College of Medicine, Bucheon, Gyeonggi 420-767, Republic of Korea

**Keywords:** *BRAF*, papillary thyroid carcinoma, peptide nucleic acid, sequencing

## Abstract

The *BRAF* point mutation is the most common genetic event in papillary thyroid carcinoma (PTC), occurring in 29–69% of such tumors. The V600E mutation accounts for up to 95% of all *BRAF* mutations. Therefore, the majority of diagnostic assays have been developed to detect only the V600E mutation of the *BRAF* gene. A peptide nucleic-acid (PNA)-clamp quantitative polymerase chain reaction (qPCR) was developed to detect the V600E mutation and other mutations in the *BRAF* gene. In this study, a 3-bp deletion mutation (c.1799_ 1801delTGA) was detected in a subject with a PTC by PNA clamp qPCR, in contrast with the results of allele-specific (AS)-PCR. The mutant allele was not detected by AS-PCR, but was detected using PNA-clamp PCR. The atypical 3-bp deletion mutation (c.1799_1801delTGA) was identified by confirmatory PCR combined with sequencing. The conversion of codons 600 (GTG) and 601 (AAA) into a single codon (GAA) resulted in the insertion of a glutamic acid residue into the activation segment of the B-raf protein (p.V600_K601delinsE). In cases where PTC is highly suspected but no mutation is detected by AS-PCR specific for V600E, PNA clamp qPCR, which is complementary to other sequencing methods, should be performed in order to detect other mutations in the *BRAF* gene.

## Introduction

Various oncogenes and tumor suppressor genes regulate the development and progression of papillary thyroid carcinoma (PTC). However, few of these have been identified to date (1). Numerous tumors have a thymine (T) to adenine (A) transversion at nucleotide position 1799 (T1799A) of the *BRAF* gene, resulting in a valine (V) to glutamate (E) substitution at residue 600 (V600E) (2,3). This *BRAF* point mutation is the most common genetic event in PTC, occurring in 29–69% of such tumors (4). The incidence rate of *BRAF* V600E in Korean patients with PTC is relatively high compared with that in other countries (5–7). Few other *BRAF* mutations have been documented. Therefore, the majority of diagnostic assays, including allele-specific (AS) amplification and quantitative polymerase chain reaction (qPCR) assays, have been developed to detect only the V600E mutation of the *BRAF* gene.

A peptide nucleic acid (PNA) clamp qPCR assay was developed to detect not only the V600E mutation but also other mutations in the *BRAF* gene. This method is based on the use of a PNA probe that inhibits the amplification of the wild-type allele while enabling the extension of mutant alleles. In this study, a 3-bp deletion mutation (c.1799_1801delTGA) was identified in a subject with a PTC by PNA clamp qPCR, in contrast with the results obtained by AS-PCR, which did not detect the mutation.

## Case report

A 69-yr-old female patient was transferred from the local clinic to Soonchunhyang University Bucheon Hospital (Bucheon, Korea) in order to evaluate thyroid masses detected with thyroid ultrasonography. The largest mass was isoechoic, and measured ~0.9×1.2 cm, with a benign nature among multiple masses in both lobes. A smaller nodule sized mass of 0.7×1.0 cm located in the lower pole of the left lobe was hypoechoic, ill-defined and solid, and suspected to be malignant. Fine-needle aspiration cytology and *BRAF* mutational analysis were performed on the suspicious lesion.

A thyroid aspiration sample was obtained from the patient after obtaining informed consent. Genomic DNA was isolated from the sample using the QIAamp^®^ DNA Mini kit (Qiagen, Chatsworth, CA, USA). AS-PCR was performed to detect mutant alleles in the *BRAF* gene using the Anyplex™ BRAF V600E Real-time Detection kit (Seegene, Seoul, Korea) according to the manufacturer’s instructions. PCR was performed in a total volume of 20 μl containing 50 ng DNA and 15 μl PCR master mix. PCR cycling was performed at 95°C for 15 min followed by 15 cycles at 95°C for 15 sec and 60°C for 30 sec, and 35 cycles at 95°C for 30 sec and 60°C for 32 sec. The fluorephores, 6-carboxyfluorescein (FAM) probe was designed to hybridize completely to the V600E allele of the *BRAF* gene. The Quasar 670 probe (Seegene) was used for hybridization with an internal control. The threshold cycle (Ct) was automatically calculated from PCR amplification plots, in which fluorescence was plotted against the number of cycles. If the Ct of the FAM probe was <33 and the Ct of Quasar 670 was <30, the *BRAF* mutation was determined to be positive, according to the instructions. A Ct value >33, characteristic of the wild-type sequence, was calculated in this study.

**Figure 1:**
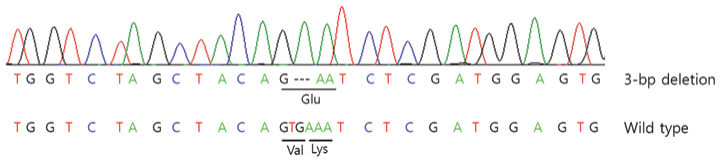
Mutation analysis of the *BRAF* gene from a patient with papillary thyroid carcinoma. Direct sequencing of exon 15 following mutant enrichment with 3′-modified oligonucleotide polymerase chain reaction (PCR) shows a 3-bp deletion (arrow) from nucleotide positions 1799 to 1801 (c.1799_1801delTGA).

In order to investigate the possibility of other *BRAF* mutations, PNA clamp qPCR was performed using the PNA Clamp BRAF Mutation Detection kit (Panagene Inc., Daejeon, Korea) according to the manufacturer’s instructions. PCR was performed in a total volume of 20 μl containing 50 ng DNA, 13-μl real-time SYBR-Green PCR master mix and each of the primers and PNA probes for codon 600. The primers and PNA probes for codon 600 were included in the PNA Clamp BRAF Mutation Detection kit. The PCR control lacked a PNA probe and contained the wild-type template. PCR cycling conditions were performed at 94°C for 5 min followed by 40 cycles at 94°C for 30 sec, 70°C for 20 sec, 63°C for 30 sec and 72°C for 30 sec, and a final extension at 72°C for 5 min. The PNA probe was designed to hybridize completely to the wild-type *BRAF* allele. PNA probe hybridization securely inhibited amplification of the wild-type *BRAF* allele, whereas the PNA/mutant allele hybrid was unstable due to a base pair mismatch and therefore did not inhibit extension by Taq polymerase. The Ct was automatically calculated from PCR amplification plots in which fluorescence was plotted against the number of cycles. The change in Ct values (ΔCt) = Ct value of the sample - the Ct value of the control. A higher ΔCt value meant that the mutant was amplified more efficiently. A cut-off value of 2.0 was used to determine the presence of mutant DNA. The ΔCt was of the sample from the patient was 8.39, suggestive of a definite *BRAF* mutation, in contrast with the AS-PCR results. In order to confirm this observation, PCR and sequencing of the *BRAF* gene was performed. For PCR, two generic primers and a blocking primer were used to amplify exon 15 of *BRAF*. Generic primers were as follows: forward: 5′-CAG TAA AAA TAG GTG ATT TTG GTC TAG C-3′; reverse : 5′-CTG ATT TTT GTG AAT ACT GGG AAC T-3′. Blocking primers were as follows: 5′-GGT GAT TTT GGT CTA GCT ACA GTG A-3′. Exon 15 was analyzed since the majority of *BRAF* mutations, including V600E, occur within exon 15 of the *BRAF* gene. The amplification product (5 μl) was treated with 2 units shrimp alkaline phosphatase and 10 units exonuclease I (USB Corp., Cleveland, OH, USA). Direct sequencing was performed using the BigDye Terminator Cycle Sequencing Ready Reaction kit on an ABI Prism 3130 Genetic analyzer (Applied Biosystems, Foster City, CA, USA). The obtained sequences were analyzed and compared with a reference sequence (NM_004333.4). A 3-bp deletion mutation in coding nucleotides 1799 to 1801 (c.1799_1801delTGA) within the *BRAF* mutational hotspot region was identified (Fig. 1). The conversion of codons 600 (GTG) and 601 (AAA) into a single codon (GAA) resulted in the insertion of a glutamic acid into the activation segment of the B-raf protein (p.V600_K601delinsE).

A total thyroidectomy with dissection of cervical lymph nodes was performed on the patient for PTC. Pathological findings of the nodule revealed classical PTC. The gross surface of the lesions were ill-defined with a hard consistency and measured 1.2×0.9 cm in the lower pole of the left lobe. No metastasis was detected in the four resected lymph nodes. The study was approved by the ethics committee of the Institutional Review Board of Soonchunhyang University Bucheon Hospital (Bucheon, Korea).

## Discussion

In this study, a rare deletion mutation that was undetectable by AS-PCR was identified. The 3-bp deletion mutation (c.1799_1801delTGA) detected in this study has been reported in other studies concerning thyroid cancer and other malignancies (8,9). Rare *BRAF* mutations in PTC have been summarized in a previous study (10) and on an online database (www.sanger.ac.uk/genetics/CGP/cosmic). Jung *et al* reported four cases with unusual BRAF mutations (excluding V600E) in 1,041 patients with PTC (11). In the present study, another case of a *BRAF* deletion mutation in PTC was identified. In this case, the pathology of the carcinoma was of the classic papillary type.

Numerous laboratories perform screenings specifically for *BRAF* V600E, which is the most common mutation in PTC, using AS-PCR or other amplification methods. However, these initial screenings may consider a deletion or other mutations in exon 15 of the *BRAF* gene (excluding the thymidine to adenine substitution at nucleotide position 1799) as the wild-type *BRAF* gene, as described in this study. Therefore, the use of other analytical methods, which are capable of detecting a variety of atypical mutations in the *BRAF* gene, is important for the evaluation of the biological characteristics, pathological features and clinical behaviors of tumors harboring *BRAF* mutations. There are a number of moderate to highly sensitive PCR methods for mutant amplification that block normal allele amplification, including PCR clamping mediated by PNA or locked nucleic acid (LNA), amplification refractory mutation system PCR and thermostable restriction endonuclease-mediated selective PCR (12). PCR clamping by PNA is a simple and sensitive mutant amplification technique, based on inhibition of the amplification of the normal allele due to the strong binding of the PNA probe to the wild-type allele. If a *BRAF* mutant is present, the binding capacity of the PNA probe for the mutant allele is weaker than for the wild-type allele, as nucleotides of the mutant allele do not match the PNA probe. Consequentially, a *BRAF* mutation may be selectively amplified; therefore, this method is useful for detecting mutant alleles, even if they are present at low concentrations in the tissue. This analytical technique is easier to design, compared with previous methods, and may be applicable to the clinical and diagnostic screening of PTC. The mutant amplification efficiency of PNA-clamp qPCR is comparable to that of PCR combined with sequencing when the reaction conditions are optimized (13). In the present study, the sequencing method successfully detected an atypical *BRAF* deletion mutation.

In conclusion, PNA-clamp qPCR was used to detect a rare 3-bp deletion in coding nucleotides 1799 to 1801 in the *BRAF* gene in a PTC patient. In cases where PTC is highly suspected, but no mutation is detected using AS-PCR specific for V600E, the use of PNA clamp qPCR (which is complementary to the sequencing method) to detect other mutations in the *BRAF* gene is recommended.
